# Influenza Vaccination Coverage, Motivators for, and Barriers to Influenza Vaccination among Healthcare Workers in Wroclaw, Poland

**DOI:** 10.3390/ijerph19031586

**Published:** 2022-01-30

**Authors:** Michał Jacek Jędrzejek, Agnieszka Mastalerz-Migas

**Affiliations:** Department of Family Medicine, Wroclaw Medical University, W. Syrokomli 1, 51-141 Wrocław, Poland; agnieszka.mastalerz-migas@umed.wroc.pl

**Keywords:** influenza, vaccine coverage, healthcare workers, vaccine hesitancy, vaccine objections

## Abstract

Background: Influenza vaccination, as a key element of control activities intended to prevent nosocomial influenza transmission, is recommended each year for all healthcare workers (HCWs). The objectives were to determine the rate of influenza vaccination and to identify reasons for receiving or declining the influenza vaccine among HCWs in the 2018/19 and 2019/20 influenza seasons. Methods: This study is a cross-sectional observational study carried out between January and March 2020, in 2 hospitals and 15 primary health-care settings (PHCS) in Wroclaw (Poland). Results: A total of 165 questionnaires were completed. The majority of participating HCWs were female—137 (83.0%), and, by profession, the majority were physicians 92 (55.8%). Influenza vaccination coverage was 61.2% in 2019/20, and 47.9% in the 2018/19 season for all participants. Participants who were male, physicians and personnel from PHCS were more frequently vaccinated in both seasons. According to the statistical analysis, physicians were more likely to receive vaccinations than nurses (*p* < 0.01), as were HCWs who had been vaccinated in the previous season (*p* < 0.001). Conclusion: The identified barriers were mainly caused by misconceptions (fear of vaccine adverse effects and perception of not being at risk/no need to get vaccinated) and an organizational barriers (lack of time). These findings may prove useful for designing immunization campaigns to tailor strategies to reach specific groups.

## 1. Introduction

The World Health Organization (WHO) and the US Advisory Committee on Immunization Practices (ACIP) both recommend that healthcare workers (HCWs) should receive influenza vaccination annually because they care for persons at high risk of influenza-related complications [1,2]. Vaccination of HCWs is an important strategy for reducing the transmission of influenza from healthcare staff to their patients, and therefore reducing patient morbidity and mortality [3,4], increasing patient safety and reducing work absenteeism among healthcare workers [5,6].

Compliance with recommendations on influenza vaccination is known to be low. In general, the rate of influenza vaccination among HCWs rarely exceeds 40% [7,8]. In European countries, the median of vaccination coverage rate (VCR) among HCWs remains at around 25% with a wide variation among countries (for example, from 5% for Poland to >50% for the UK) [9]. Since influenza vaccine uptake among HCWs still remains low, more information is needed about barriers to influenza vaccination in HCWs. Refusals to vaccinate can be attributed to uncertainty about the effectiveness and safety of the vaccine [10,11]. A large number of diverse reasons for low vaccine uptake by HCWs have been addressed in the literature [8,12,13,14]. Understanding these barriers is important as it reveals the complexity of the situation and is essential for increasing the levels of compliance with vaccination recommendations.

Results of a literature review suggest that there were no exhaustive and comprehensive data on attitudes towards influenza vaccine uptake by HCWs in Poland. Only two surveys have been conducted—the first was carried out 10 years ago in Warsaw (the capital city of Poland) and revealed the VCR for hospital personnel to be at approximately 20% [15]; the second one was a national cross-sectional survey with participation by 500 physicians involved in the qualification and administration of childhood vaccines (81% were pediatricians, and the remaining 19% were family doctors), conducted from June to July 2017, with a result of 62% of seasonal influenza VCR [16]. In addition, the VCR of the general population in Poland is extremely low (3.5%) [17].

While influenza vaccination has variable and moderate efficacy [18], given the current epidemiological situation of the COVID-19 pandemic, it seems appropriate to make every effort to reduce the burden of influenza virus-induced infections on the health system and help protect limited healthcare resources. Due to very high variability of VCR and its low level among HCWs in Poland, there is an urgent need for extending the database, especially with local analyses. A cross-sectional observational survey was conducted in Wroclaw (one of the major cities of Poland), between January and March 2020. The objective of this study was to analyze vaccination rates and motivators for, and barriers to, influenza vaccination among the participating HCWs. The results may prove useful for designing immunization campaigns to tailor strategies to reach specific groups.

## 2. Materials and Methods

### 2.1. Study Design

To draw a study sample, a register of public primary healthcare settings (PHCS) and hospitals in Wrocław obtained from the national health services was used (a list of public healthcare facilities is available online in an open-access mode). Due to organizational limitations, it was planned to include nineteen PHCS (12%) pre-selected from all 153 PHCS in Wrocław using systematic sampling, and 3 hospitals were pre-selected from 6 main facilities using purposive sampling. All preselected PHCS and 2 of 3 hospitals accepted the invitation to participate in this study, 1 hospital rejected the invitation. Five or four wards per hospital were selected to participate, mainly internal-medicine and pediatric wards (patients at high risk of influenza). All personnel of the selected PCHS and hospital wards were set as the target population.

Recruitment was performed by inviting all medical and non-medical staff to participate during personal visits of the principal investigator in selected healthcare facilities. Participation in the study was voluntary. Approval for distributing the questionnaire was obtained from the board of each healthcare facility participating in the survey. After receiving written information about the study and a brief oral description of the aim of the study, written informed consents were obtained from all of the participants before entering the study, and then the participants received self-administered standardized questionnaires to complete.

The study was terminated prematurely due to the COVID-19 epidemiological situation. Eventually, HCWs from fifteen selected PHCS (10% of all PHCS in Wroclaw) and part of selected hospital units (endocrinology, hematology, oncology, angiology and rheumatology units from the University Clinical Hospital and a pediatric intensive care unit from another multidisciplinary hospital) participated in the study.

### 2.2. Study Questionnaire

The anonymous self-administered questionnaire was composed of two sections. The first section included socio-professional variables, such as demographic details (gender, age), occupational group, type of healthcare facility (primary healthcare settings/hospital), and years of experience. Occupational groups were categorized as physicians, nurses, allied medicals (for example, physiotherapists and laboratory diagnosticians) and non-medical staff (administration, cleaning and other support staff). Medical students were excluded from participating in this study. The second section assessed self-reported uptake of influenza vaccination in the 2018/19 and 2019/20 seasons and potential motivators for and barriers to influenza vaccination in the survey or previous year (multiple choice responses or a free text field for another answer). Only the personnel who were vaccinated were asked about motivators for vaccination (e.g., self-protection, protection of family, protection of patients) and only the personnel who were not vaccinated were asked about barriers to vaccination (e.g., low effectiveness of the vaccine, lack of time, fear of side effects, no need for protection).

### 2.3. Outcome Measures

There were two outcome measures in this study: (1) the current and previous influenza vaccination status of HCWs and (2) the assessment of the determinants and the reasons for (not) being vaccinated against influenza.

### 2.4. Statistical Analysis

Upon completion of data collection, the data were coded into categorical variables and double-checked. Descriptive statistics were generated for all survey items. The main analysis was a comparison of vaccinated and not vaccinated respondents, defined by their self-reported influenza vaccination status during the 2018/19 and 2019/20 influenza seasons. Categorical variables were compared using Pearson’s χ^2^ tests of association with Yates’ continuity correction and correspondence analyses were used. The significance level was set at *p* = 0.05. The statistical analyses were performed using R version 3.6.3 statistical software (R Foundation for Statistical Computing, Vienna, Austria).

## 3. Results

### 3.1. Study Population

A total of 165 questionnaires were completed (response rate 82.5%). Nearly 56% of respondents worked in PHCS. There were 92 (55.8%) physicians and 43 (26.1%) nurses in total. Females accounted for 83.0% of respondents. Approximately 60% of the respondents were aged over 40. Their years of experience ranged from 1 to 43 years with a median of 15 years. There were no significant statistical differences between survey HCWs from PHCS and hospitals in terms of their questionnaire-based socio-professional determinants (*p* > 0.05). The characteristics of the study participants are presented in Table 1.

### 3.2. Vaccination Rate and Socio-Professional Determinants

Influenza vaccination coverage was 61.2% (101/165) in 2019/20, and 47.9% (79/165) in the 2018/19 season for all participants (Table 1). The difference between the vaccination rates of hospital-based personnel (60.8% and 39.2%) and personnel from PHCS (61.5% and 54.9%) was statistically insignificant in the two analyzed influenza seasons, 2019/20 and 2018/19 respectively (*p* > 0.05). The results revealed that gender, age and job experience had no statistically significant effect on the vaccination rate of participants with a *p* > 0.05 with Yates’ continuity correction in both influenza seasons (Table 1). The 2018/19 and 2019/20 vaccination rates by profession ranged from 34.9% to 41.9% for nurses and from 63.0% to 78.3% for physicians respectively (*p* < 0.01). A correspondence analysis has confirmed that there is a correlation between the following pairs of variables: (a) physicians were more likely to receive influenza vaccination and (b) nurses were less likely to receive influenza vaccination in the two analyzed influenza seasons. Influenza vaccine uptake in 2019/20 was also strongly associated with the status of 2018/19 influenza immunization (*p* < 0.001), namely the previous history of immunization was positively correlated with influenza vaccine uptake in the survey season.

### 3.3. Reasons for Receiving or Rejecting Vaccination

Three main motives and seven main barriers were identified to influenza vaccination in the survey participants (Table 2). Self-protection (98.1%) was the main reason for vaccination, whereas 82.2% of vaccinated HCWs reported receiving vaccination to protect their families and 65.4% to protect their patients. The same motivator structure was reported regardless of the type of healthcare facility (PHCS/hospital) and profession (physicians/nurses), i.e., without statistically significant differences (*p* > 0.05). Reasons for refusing a vaccine (reported for at least one influenza season) were provided by 92 participants. The top three identified barriers to vaccination were: lack of time (30.4%), fear of side effects (15.2%) and perception of not being at risk/no need to get vaccinated (10.9%). HCWs from PHCS more frequently reported fear of side effects (20%) and belief that they do not need to be vaccinated (17.8%) than hospital personnel (10.6% and 4.3% respectively). By contrast, laziness was a significant barrier to vaccine uptake among hospital-based HCWs (14.9%) compared to HCWs from PHCS (4.4%). Moreover, lack of time and laziness were the most common reasons for refusing the vaccine among physicians (45.0% and 15.0% respectively) in contrast to nurses (25.0% and 0% respectively). On the other hand, nurses reported fear of side effects (17.9%) and fear of injection (10.7%) more frequently when compared to physicians (5.0% and 2.5% respectively).

## 4. Discussion

Compared to the data available for Poland, our study showed a relatively high vaccination rate among HCWs in Wroclaw (in the range of 47.9 to 61.2% for two influenza seasons). Despite high consistency with the latest survey from the National Institute of Public Health—National Institute of Hygiene, which reports VCR at 62% [16], the present authors attempted to explain this result. This may be a selection or response bias, due to the potential lack of compatibility with the general population of Wroclaw HCWs and voluntary participation in the study—together it could lead to an overestimation of VCR. Nonetheless, data from recent surveys (conducted after 2010) among HCWs in hospital and PHCS demonstrate a similar level of influenza vaccine uptake. For example, a Spanish survey shows VCR at 50.7% among physicians and nurses working in PHCS (*n* = 1749; season 2011/12) [19], data from Arar City (Saudi Arabia) demonstrate VCR at 55.9% among HCWs from PHCS (204 participants, season 2017/18) [20] and an influenza vaccination rate of 55% among HCWs in a German university hospital (677 participants, season 2014/15) [21]. It is worth emphasizing that VCR is a variable over time (for example, VCR from 54.5% to 88.3% in 2012/13–2014/15 seasons in a study from King Abdullah University Hospital [22] or VCR for Romania in 2007/08 season at 89.4% and 29.4% in 2014/15 season in longitudinal data from the European Union [9]). Moreover, the substantial number of vaccinated physicians in our study can be explained by the fact that the study participants were specialists who care for patients at high risk of influenza and therefore tend to be more aware of the importance of regular vaccinations compared to other specialists. On the other hand, protection of patients was a motivator for vaccine uptake in the third place for all participants (65.4%), as well as for physicians (69.2%).

The current survey shows that nurses were less frequently vaccinated compared to physicians and this difference was statistically significant (*p* < 0.01) for both of the analyzed influenza seasons (Table 1). Our data are consistent with findings from other studies in that vaccine uptake is significantly lower for nurses than physicians [23,24,25,26], including one Polish survey [15]. To explain this correlation, it is worth highlighting findings from other studies demonstrating that physicians in general had a higher level of knowledge about influenza and influenza vaccines and therefore perhaps they were more likely to receive vaccine [23,26,27]. For example, in an Israeli study, more than half of nurses (53.5%) reported that vaccination per se can cause flu (vs 35.9% physicians) [23] and in a German study—19.2% of nurses (vs 0% of physicians, *p* < 0.001) [27], which of course is not true (subunit vaccines containing only hemagglutinin are commonly used). Similarly, the proportion of HCWs who perceived the vaccine to be harmful was higher among nurses and allied health professionals than among doctors in a hospital-based Israeli study [28]. A qualitative Swiss study conducted among nurses could be helpful in explaining this phenomenon, as it demonstrated that the main barriers to vaccination include fear of side effects, doubt about the effectiveness of vaccination and the will to autonomously make decisions about one’s body and health, as well as distrust of medical environment and research results [29]. Targeted educational strategies may be needed to resolve misconceptions among nurses. This observation seems to be confirmed by the results of a Korean study concerning the effectiveness of educational campaigns—after an intensive campaign promoting influenza vaccination among hospital staff, an increase in the vaccination rate from 21% to 92% was observed among nursing staff in 4 years (2000–2004) [30]. This is also confirmed by a Chinese study—nurses who underwent vaccination training in the preceding 5 years were statistically significantly more often vaccinated against influenza [31].

Many studies show a positive correlation with the male gender [20,21,32,33,34,35,36] or age over 40 years [22,26,33,35,37] on influenza vaccine uptake among HCWs. However, not all studies demonstrated that gender or age were significant predictors of vaccination [28,38]. The results of the current study showed that gender and age were not statistically significant for the vaccination rate of participants in both influenza seasons. Nonetheless, it is worth highlighting that both gender and age were statistically significant in the 2019/20 influenza vaccine uptake using “pure” χ^2^ test (*p* < 0.05), but according to more conservative Yates’ continuity correction, these correlations had no statistical significance with *p* > 0.05. This could result from the small size of the sample. Similarly, job experience had no statistically significant effect on the vaccination rate, as opposed to the results of other studies [20,22,36,37].

Another finding in our study illustrated that a previous history of immunization was positively correlated with the influenza vaccine uptake in the participants of this survey (*p* < 0.001), which is highly consistent with the results of many studies [10,23,39,40,41].

It is worth highlighting that interventions based on identified factors are useful for designing immunization campaigns to tailor strategies to reach specific groups. Self-protection and protection of family/friends were the most common reasons to accept influenza vaccination in the current survey—98.1% and 82.2% respectively. 65% of the vaccinated HCWs reported a reduction in virus transmission to their patients as motivation for vaccination. These reasons are highly compatible with other studies based on larger study samples, for example, 95.0% of vaccinated HCWs from Saudi Arabia (512 hospital-based participants) [40], 92.5% of vaccinated HCWs from Israel (275 participants from PHCS) [23] and 92.2% of vaccinated HCWs from a German survey (four thousand workers from a university hospital) [27] indicated self-protection from influenza as a motivation to immunize, whereas the protection of patients motivated 64.2% of HCWs from an Arab survey [40] and 54.7% of German HCWs [27]. Similar results were reported among HCWs in many other studies [10,11,24,26,32].

The identified barriers were mainly caused by organizational barriers (lack of time), and misconceptions (fear of vaccine adverse effects and perception of not being at risk/no need to get vaccinated). In general, the barriers to influenza vaccine uptake by HCWs found in this study were similar to those reported in the literature. For example, not vaccinated nurses from a German study reported fear of adverse effects and fear of injection more often than physicians [27], whereas lack of time was more frequent for physicians than for nurses in an Israeli study [28]. Moreover, 31.8% and 17.3% of not vaccinated HCWs from an Arab study reported “lack of time” and were concerned about side effects of the vaccine [42] compared to 30.4% and 15.2% of not vaccinated HCWs from the present survey respectively. Similarly, lack of time was reported by 33% of French HCWs [43] while 14% of Italian hospital-based HCWs were concerned about side effects [34]. In addition, 24% of hospital-based physicians from a Polish study reported lack of time as the most common reason for not being vaccinated [15] (compared to 18% hospital-based physicians from the present survey; data not shown). According to Hofmann et al., well-planned campaigns should be useful for increasing vaccination coverage, especially among physicians [44]. It is worth highlighting that lack of time was the main barrier to vaccine uptake among HCWs from the present survey (30.4%), especially among physicians (45.0%). Moreover, the majority of immunized HCWs (77.6%) were vaccinated as part of occupational vaccinations and more than half of not vaccinated HCWs (51.1%) declared immunization if vaccination was to be organized at their workplaces (data not shown).

It is emphasized in the literature that activities aimed at increasing the level of influenza vaccination among healthcare professionals must take into account the complexity of the problem, including numerous vaccination conditions (individual, psychological, socio-cultural, ethical and organizational factors) [45]. Experience so far shows that misconceptions (e.g., fear about vaccine side effects and no perception of personal risk of influenza infection) can be reduced/corrected thanks to a well-planned educational program, taking into account the psychological and socio-cultural specificities of the recipients, and access to vaccination can be improved by offering free workplace vaccinations. As the described experiences show, strategies combining various interventions (a multi-component strategy) are more effective than single-component interventions. The necessity to take complex actions is evidenced by, inter alia, the fact that educational campaigns alone do not significantly increase the vaccination rate, and simply offering free influenza vaccination is not enough [45]. The effectiveness of active promotional and educational activities has been repeatedly confirmed, both in reports from Europe, the USA and Japan (the offer of free workplace vaccinations in conjunction with an educational campaign conducted simultaneously) [18,45,46]. Some researchers, paying attention to the differences in the level of vaccination and the declared barriers between different HCWs occupational groups, suggest the need for separate strategies [44], which is also confirmed by the results of this study. It is postulated that the activities dedicated to nursing staff should be based on educational campaigns aimed at reduction/correction of misconceptions, in turn, promoting campaigns dedicated for physicians should be based on easy and free vaccination on site. At this point, it is worth highlighting that HCWs reported a preference for educational messages which should be: targeted at HCWs (not general messages), based on robust evidence and which ought to address specific concerns abound vaccine effectiveness and risks [18]. It should be borne in mind that during influenza vaccination campaigns it is important to focus also on personal benefits for HCWs themselves, since self-protection and protection of family/friends were the most common reasons for the acceptance of influenza vaccination, not only in the current survey.

### Strength and Limitations

This is the first study to assess the rate of influenza vaccination among Wroclaw HCWs and one of the few in Poland in the last 10 years. In addition, HCWs from both types of healthcare facilities were included, enabling their direct comparison. Moreover, this study identified motivating factors and barriers to influenza vaccine uptake similarly to a variety of studies around the world.

There are several limitations to this study. Selection bias is possible and due to this fact, the study sample may not be fully representative of HCWs in Wroclaw. First, participation in the study was on a voluntary basis and it is possible that motivated (and vaccinated) HCWs were more likely to participate and to complete the survey than their not vaccinated colleagues (this result was observed in an Israeli study [28]). In addition, the possibility to generalize the findings from this study is limited because the survey population cannot be compared with the general population due to the lack of a full list of HCWs from all Wroclaw PHCS and hospitals. On the other hand, a similar strategy of study population sampling (selection based on the list of facilities, not on a list of HCWs population) was used by many other authors in their research, for example by Boey et al. (2018) [36], Lee et al. (2017) [31], Abu-Gharbieh et al. (2010) [42] and Dominguez et al. (2013) [19]. However, we cannot exclude the possibility that there are other unknown differences between respondents and non-respondents. It is worth noting that recruitment by providing all medical staff with a paper questionnaire with an invitation to participate may be characterized by a low response rate (e.g., 25% [31], 31% [21], 32.5% [43]) as in the case of studies using telephone interviews (19% [16]) or anonymous online questionnaires (17.9% [36], 36.2% [19]). For these reasons, a personal invitation to participate with an ad hoc possibility of explaining the aim and course of the study seems to be more appropriate. Self-report is another possible limitation of the study. Influenza vaccination status was reported by the respondents themselves, which was not subject to independent verification, therefore the accuracy of the responses depended only on each respondent’s willingness to admit they had (not) been vaccinated (although one study demonstrated a good level of sensitivity and specificity of self-reported HCWs influenza vaccination with vaccination records [47]). In addition, this survey was conducted mainly among HCWs from one hospital and only 10% of PHCS in Wroclaw during one influenza season—it reflects the current influenza immunization status and does not describe changes over time. Actually, the sample size of the present study, i.e., 165, may not be large enough to have high statistical power to identify small-to-moderate associations. There is a significant need to conduct more extensive research on representative population of Wroclaw healthcare personnel.

## 5. Conclusions

HCWs from the present study decline vaccination because of lack of time, fear about vaccine side effects and no perception of personal risk of influenza infection. The identified barriers are mainly based on misconceptions and the lack of coordinated vaccination action. These determinants can be used to fine-tune the objectives of the campaign and to determine the best strategy. Misconceptions can be reduced through a well-planned educational program which should be aimed at correcting misconceptions about vaccine safety and effectiveness as well as promoting the involvement of HCWs. Our findings prove that during influenza vaccination campaigns it is important not only to focus on patient values, but also on personal benefits for HCWs themselves. In nursing staff, campaigns promoting influenza vaccination should focus on reducing fear of adverse events and increasing knowledge on influenza and the benefits of vaccination. Well-planned campaigns with an extended offer of easy and free vaccination on site (e.g., mobile vaccination teams) should be useful for increasing vaccination coverage, especially among physicians. We plan to use these findings in our influenza vaccination program and target populations with low vaccination rates for more intense intervention.

## Figures and Tables

**Table 1:** Characteristics of the study participants and the 2018/19 and 2019/20 influenza vaccination rates.

Characteristics	Total ^a^		PHCS ^a^		Hospital ^a^		*p*-Value *	2018/19 Influenza Vaccinated ^a^		2018/19 Influenza VCR	*p*-Value *	2019/20 Influenza Vaccinated ^a^		2019/20 Influenza VCR	*p*-Value *
Gender															
Female	137	(83.0)	78	(85.7)	59	(79.7)		63	(79.7)	46.0%		79	(78.2)	57.7%	
Male	28	(17.0)	13	(14.3)	15	(20.3)	0.418	16	(20.3)	57.1%	0.385	22	(21.8)	78.6%	0.064
Age group (years)															
≤40	71	(43.0)	33	(36.3)	38	(51.4)		32	(40.5)	45.1%		50	(49.5)	70.4%	
>41	94	(57.0)	58	(63.7)	36	(48.6)	0.074	47	(59.5)	50.0%	0.638	51	(50.5)	54.3%	0.051
Occupation															
Physicians	92	(55.8)	47	(51.6)	45	(60.8)		58	(73.4)	63.0%		72	(71.3)	78.3%	
Nurses	43	(26.1)	27	(29.7)	16	(21.6)	0.277 ^d^	15	(19.0)	34.9%	0.004 (χ^2^ = 8.26) ^d^	18	(17.8)	41.9%	<0.001 (χ^2^ = 15.88) ^d^
Allied medical staff ^b^	11	(6.7)	5	(5.5)	6	(8.1)		1	(1.3)	9.1%		2	(2.0)	18.2%	
Nonmedical staff ^c^	19	(11.5)	12	(13.2)	7	(9.5)		5	(6.3)	26.3%		9	(8.9)	47.4%	
Work experience (years)															
≤10	65	(39.4)	30	(33.0)	35	(47.3)		27	(34.2)	41.5%		44	(43.6)	67.7%	
>11	100	(60.6)	61	(67.0)	39	(52.7)	0.087	52	(65.8)	52.0%	0.248	57	(56.4)	57.0%	0.225
2019/20 influenza immunization															
Yes	101	(61.2)	56	(61.5)	45	(60.8)		73	(92.4)	72.3%		-	-	-	-
No	64	(38.8)	35	(38.5)	29	(39.2)	0.948	6	(7.6)	9.4%	<0.001 (χ^2^ = 59.62)	-	-	-	-
2018/19 influenza immunization															
Yes	79	(47.9)	50	(54.9)	29	(39.2)		-	-	-	-	73	(72.3)	92.4%	
No	86	(52.1)	41	(45.1)	45	(60.8)	0.063	-	-	-	-	28	(27.7)	32.6%	
Place of work															
PHCS	91	(55.2)	-	-	-	-	-	50	(63.3)	54.9%		56	(55.4)	61.5%	
Hospital	74	(44.8)	-	-	-	-	-	29	(36.7)	39.2%	0.063	45	(44.6)	60.8%	0.948

PHCS—primary health-care settings, VCR—vaccination coverage rate. * *p*-value for Pearson’s χ^2^ tests of association. ^a^ Values are presented as *n* (%). ^b^ Physiotherapists, laboratory diagnosticians. ^c^ Administrative, cleaning and supporting staff. ^d^ The Pearson’s χ^2^ test was calculated only for a group of physicians and nurses due to the small number of other categories.

**Table 2:** Motivators for, and barriers to seasonal influenza vaccination in the survey participants (reported for at least one influenza season).

Motivators/Barriers	Total	Place of Work		Occupation	
		PHCS	Hospital	Physicians	Nurses
Motivators for vaccination	*n* = 107	*n* = 60	*n* = 47	*n* = 78	*n* = 18
Self-protection	98.1% (105)	96.7% (58)	100.0% (47)	98.7% (77)	94.4% (17)
Protection of family/friends	82.2% (88)	76.7% (46)	89.4% (42)	85.9% (67)	61.1% (11)
Protection of patients	65.4% (70)	61.7% (37)	70.2% (33)	69.2% (54)	50.0% (9)
		*p*-value = 0.909		*p*-value = 0.723	
Barriers to vaccination	*n* = 92	*n* = 47	*n* = 45	*n* = 40	*n* = 28
Lack of time	30.4% (28)	31.1% (14)	29.8 (14)	45.0% (18)	25.0% (7)
Fear of vaccine adverse effects	15.2% (14)	20.0% (9)	10.6% (5)	5.0% (2)	17.9% (5)
Perception of not being at risk/no need to get vaccinated	10.9% (10)	17.8% (8)	4.3% (2)	10.0% (4)	7.1% (2)
Laziness	9.8% (9)	4.4% (2)	14.9% (7)	15.0% (6)	0.0% (0)
Belief that the vaccine is not effective	8.7% (8)	8.9% (4)	8.5% (4)	5.0% (2)	3.6% (1)
Contraindications	5.4% (5)	6.7% (3)	4.3% (2)	5.0% (2)	10.7% (3)
Fear of injection	4.3% (4)	4.4% (2)	4.3% (2)	2.5% (1)	10.7% (3)
		n/a		n/a	

*p*-value for Pearson’s χ^2^ tests of association; n/a—not applicable. PHCS—primary healthcare setting. Values are presented as % (*n*). NOTE: Adds up to more than 100% because of multiple responses.

## Data Availability

The study is registered on ClinicalTrials.gov, accessed on 20 December 2021 (NCT04223544).
